# Supplementary material for: Causal Relationship between Aging and Anorexia Nervosa: A White-Matter-Microstructure-Mediated Mendelian Randomization Analysis
Source: Biomedicines. 2024 Aug 16;12(8):1874. doi: 10.3390/biomedicines12081874 (PMC11351342; doi:10.3390/biomedicines12081874)
Supplement: Supplementary file 1 [file biomedicines-12-01874-s001.zip › Supplementary Figure/Figure S8/Leaveoneout plot of MR analysis for TL to ubm-b-2048.pdf]

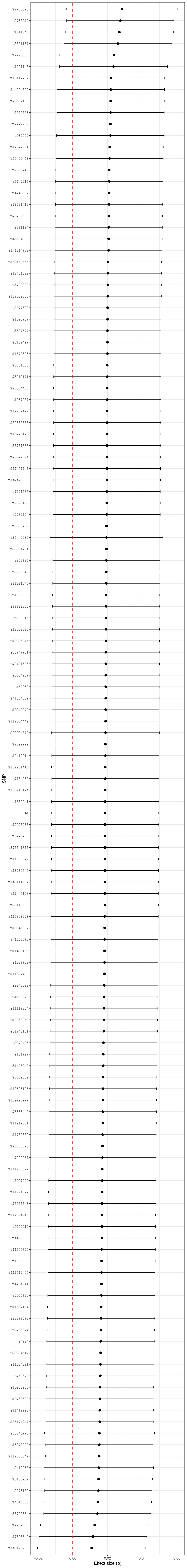
rs7705526
rs2763979
rs611646
rs3891167
rs7790856
rs1291143
rs10112752
rs144204502
rs28502153
rs6669563
rs7772289
rs932002
rs17677991
rs59409453
rs2538745
rs5742915
rs4743037
rs73581419
rs73730598
rs871134
rs45604339
rs141214782
rs150150565
rs12451892
rs6790988
rs182059586
rs2977608
rs1023767
rs6587577
rs8102497
rs11579626
rs6881568
rs76219171
rs75664430
rs1957937
rs12932179
rs139669835
rs10773176
rs66731853
rs28577594
rs117407747
rs142426306
rs7221585
rs9398196
rs2282764
rs6536702
rs35446936
rs56061761
rs869785
rs6590343
rs77231040
rs1003322
rs77732866
rs939916
rs13062095
rs10805346
rs55747751
rs78491606
rs6054257
rs450962
rs41304832
rs10840270
rs117034449
rs202034370
rs7099229
rs12412214
rs137901416
rs7164950
rs188918174
rs1332941
All
rs12925933
rs6776756
rs376641875
rs11085072
rs13230646
rs145114957
rs17445108
rs80116508
rs116863223
rs10845387
rs41269079
rs11426156
rs1907702
rs111527438
rs9940099
rs4530278
rs11117354
rs12369950
rs61748181
rs9878436
rs131797
rs61405042
rs6659668
rs113525195
rs139795227
rs76666449
rs11212631
rs11769630
rs28363070
rs7209057
rs111950327
rs6007020
rs11991877
rs76065543
rs112394943
rs9600019
rs4498805
rs11699829
rs1985369
rs117512405
rs4731541
rs2056726
rs11557154
rs79977579
rs3785074
rs4724
rs80324517
rs11584821
rs762679
rs10905255
rs10768683
rs11412296
rs185174247
rs35640778
rs16978028
rs117630647
rs9419958
rs8105767
rs2276182
rs4616688
rs56799554
rs2967355
rs17803849
rs143190905
SNP
-0.02
0.00
0.02
0.04
0.06
Effect size (b)
